# Effect of iron oxide content and microstructural porosity on the performance of ceramic membranes as microbial fuel cell separators

**DOI:** 10.1016/j.electacta.2020.137385

**Published:** 2021-01-20

**Authors:** M.J. Salar-García, X.A. Walter, J. Gurauskis, A. de Ramón Fernández, I. Ieropoulos

**Affiliations:** a Bristol BioEnergy Centre, Bristol Robotics Laboratory, T-Block, University of the West of England, Bristol BS16 1QY, United Kingdom; b ARAID Foundation, Aragón Materials Science Institute (CSIC-Unizar), Zaragoza E-50009, Spain; c Department of Computer Technology, University of Alicante, Alicante E-03690 Spain

**Keywords:** Microbial fuel cells, Bioenergy, Ceramic membranes, Iron oxide content, Urine

## Abstract

Ceramic materials based on naturally occurring clays are a low cost and environmentally friendly alternative to commercial polymer-based membranes in bioelectrochemical systems. In this work, ceramic membranes containing different amounts of iron oxide (1.06, 2.76 and 5.75 vol.%) and sintered at different temperatures (1100, 1200 and 1300 °C) have been elaborated and tested as separators in urine-fed microbial fuel cells (MFCs). The results reveal that the presence of iron oxide in the ceramic membrane composition increases the structural porosity and reduces the pore size for the three temperatures investigated. On the other hand, it was also observed that the iron content mitigates the negative effect of the high sintering temperature on the power performance of the MFCs. In the case of the ceramic membranes sintered at 1300 °C, power output improved ca. 10-fold when the iron oxide content in the membrane increased from 1.06 up to 5.75 vol.% (30.9 and 286.6 µW, respectively). Amongst the different combinations of iron phase content and sintering temperatures, the maximum power output was obtained by MFCs working with separators containing 5.75 vol. % of iron oxide and sintered at 1100 °C (1.045 mW). Finally, the system was stable for 65 days, which supports the long-term functionality of the different materials assessed.

## Highlights

• Suitable bioelectrochemical application of ceramic clays with varying Fe2O3 content.
• 5.75% of Fe2O3 and 1100 °C of sintering temperature allow MFCs to reach 1.045 mW.
• The Fe2O3 content mitigates the effect of porosity features on the MFC power output.
• Good stability of the MFCs working during 65 days in continuous mode.

## 1 Introduction

Microbial Fuel Cell (MFC) is an environmentally friendly technology where electroactive bacteria transform the chemical energy stored in substrates of waste nature into electricity. These devices show a double benefit over other technologies since energy production and treatment of the waste, occur simultaneously. Microorganisms in the anode perform the degradation of the organic matter contained in the substrate. The electrons released during this process flow through an external circuit to the cathode, where they combine with an electron acceptor, usually oxygen from free air via oxygen reduction reaction (ORR) [1,^2^,3]. However, the presence of a catalyst to accelerate the sluggish cathodic ORR is still needed and activated carbon is one of the most commonly used catalysts in air-breathing MFCs due to its low cost and long-term stability, compared with platinum-group metals, [4,5].

One of the most relevant factors affecting electricity generation in MFCs is the substrate, which ranges from pure compounds to complex substrates such as wastewater. The use of MFC to remove pollutant from waste streams has gained much attention since conventional treatment methods, such as the activated sludge process, have been reported to be very energy-intensive. Amongst the most common feedstock used in MFCs are industrial stream wastes from the beer industry, food processing of swine industry as well as domestic wastewater or neat urine [6,^7^,^8^,9]. In the case of urine, its abundance, high conductivity, buffering capacity and chemical oxygen demand (COD) converts this waste into a highly suitable feedstock for microbial electrochemical technologies such as MFC [10,^11^,12].

The majority of the MFC's have the anode and cathode physically separated by a selective membrane (i.e. separator), which reduces substrate losses and maintains the anaerobic condition in the anodic chamber. The high cost of commercial membranes has boosted the search for alternative materials to be used as separators in MFC's and therefore, facilitate the practical application of this technology [13]. The use of MFC technology in a remote location or developing countries for generating electricity makes it necessary to reduce the cost of the overall system. According to this approach, one of the most promising alternatives to commercial membranes is ceramic clay-based materials. Their low cost, natural availability and functional long term robustness make these cost-effective materials a feasible option for scaled-up systems [14,15]. Another advantage of using these environmentally friendly materials as membranes is that their microstructure can be easily modified by either adding other compounds to raw ceramic clay or by varying the conditions of the sintering process [16,17]. These modifications directly affect parameters such as the overall effective porosity or pore size distribution within ceramic membranes and therefore, the flux of ions through its structure, which is related to the power performance of the MFCs [18]. The presence of exchangeable cations in ceramic separators is closely related to the cation transport mechanism. The presence of exchangeable cations in raw natural clay is limited, which reduces the proton transport through this kind of membranes. So far, some minerals have been used as cation exchangers in ceramic membranes such as montmorillonite or kaolinite, amongst others. Their high cation exchangeability is related to the presence of anionic charges such as hydroxyl ions or aluminate, which increase the cation exchange capacity of the ceramic [19]. According to this approach, in 2015 Ghadge and Ghangrekar modified natural clay with montmorillonite and kaolinite in different proportions for preparing MFC separators [20]. Their results showed that natural clay blended with 20 wt.% of montmorillonite allowed MFCs to reach the highest power output (7.5 W.*m* ^−^ ^3^), 48% higher than the MFC working with the unmodified ceramic membrane. Regarding the cation exchange capacity, the presence of 20 wt.% of montmorillonite in the ceramic membrane increased this parameter up to 1.6 times compared to the bare ceramic membrane and around 2.1 times compared to commercial Nafion [20].

An alternative type of ceramic membranes named polymer-derived ceramics (PDC) obtained from organosilicon systems, such as polysiloxane, have also been tailored to improve their properties. Some of the most common procedures in order to modify the porosity of PDC membranes is by varying the pyrolysis temperature or adding materials to increase the surface area (fillers) [21]. According to this approach, Ahilan et al. (2018) studied the effect of different proton-conducting fillers such as montmorillonite and H_3_PMo_12_O_40_/SiO_2_ (PMA) and the pyrolysis temperature on the properties of PDC-based membranes [22]. Their work reported that ionic exchange capacity and cation transport number of the PDC membrane containing 10 wt.% of montmorillonite and 10 wt.% of PMA, and fired at 400 °C were 67% and 68% higher than commercial Nafion, respectively. Regarding the oxygen diffusion coefficient, this parameter reached the value of 1.79 × 10^−4^ cm^2^.*s* ^−^ ^1^ for membranes with pore size around 260 nm, which is very similar to that observed for Nafion. Their results support the potential application of these low cost modified ceramic membranes as MFC separators [22].

More recently, Ahilan et al. (2019) used multiwall carbon nanotubes (MWCNTs) and graphene oxide (GO) to functionalise PDC for being used as separators in MFCs. The authors aimed to improve the properties of the PDC membranes in terms of thermal stability and ionic conductivity, as well as reducing the oxygen transfer. Their results showed that both the mechanical stability and ion exchange capacity of the modified PDC membranes increased by the addition of GO and MWCNTs. Amongst all compositions investigated, it was found that the addition of 0.5 wt. % of GO to the PDC allowed them to increase 9 times the ionic exchange capacity of the membranes compared with the bare PDC. Moreover, the use of the modified membranes as a separator in MFCs outperformed Nafion in terms of maximum power output (7.23 W.*m* ^−^ ^3^) and coulombic efficiency (28.8%) [23].

Due to the aforementioned multiple benefits and promising properties of ceramic membranes as MFC separators, this work aims to analyse the effect of varying the iron oxide content within ceramic membranes. At the same time, a special focus was given to microstructural features such as porosity and pore size distribution and their effect on MFC power performance. To this end, the ceramic membranes had three different amounts of iron oxide (1.06, 2.76 and 5.75vol. %) and each type of membrane was sintered at three different temperatures (1100, 1200 and 1300 °C) in triplicate with a total number of 27 MFCs tested in continuous mode. The surface properties of the membranes were analysed before being tested as MFC separators and the results obtained were statistically analysed.

## 2 Materials and methods

### 2.1 Membrane synthesis

Refractory clay pastes with varying iron oxide content (Table 1) were acquired from Ceramica Collet S.A. (Spain). As received pastes were cold rolled to sheets, a punching tool was used to form green state disk-shaped geometry samples with ~2 mm thickness and 65 mm diameter. Obtained samples were dried at room temperature for 48 h and sintered using well time of 2 h at 1100, 1200 and 1300 °C with the heating and cooling ramps of 2 °C/min and 5 °C/min, respectively.

**Table 1:** Chemical composition (Vol.%) of the three types of refractory clay pastes employed.

	**Fe_2_O_3_**	**SiO_2_**	**Al_2_O_3_**	**TiO_2_**	**CaO**	**MgO**

**F1**	1.06	63.92	26.48	1.52	0.14	0.18
**F3**	2.76	57.10	30.30	1.38	0.34	0.28
**F6**	5.75	54.50	30.10	1.39	0.55	0.28

The refractory nature of clay pastes deployed, corresponding to low treatment temperatures used (max 1300 °C, dwell 2 h) during the sintering process, ensured a high control over the microstructural features. The ceramic membranes obtained had final disc geometry of 50 mm in diameter with 1.2 mm ± 0.1 mm in thickness and were grouped according to their chemical composition and sintering temperature to the following nomenclature (Table 2).

**Table 2:** Nomenclature of the ceramic membranes employed depending on the iron oxide concentrations and sintering temperatures.

Sintering temperature (°C)	Iron oxide content (vol. %)		
	**1.06**	**2.76**	**5.76**
1100	F1-T1	F3-T1	F6-T1
1200	F1-T2	F3-T2	F6-T2
1300	F1-T3	F3-T3	F6-T3

### 2.2 Microbial fuel cell construction

**Fig. 1:**
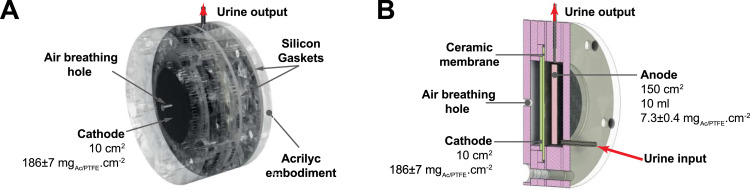
A) Isometric view of the 3D design of one MFC, from the cathodic side. B) Section view of the cylindrical MFC showing the disposition of its internal components.

The air-breathing microbial fuel cells were built by assembling laser-cut acrylic sheets (Fig. 1). These acrylic sheets were held in place by 5 bolts and separated when needed by 1.5 mm silicon gaskets to form the MFC housing. The anode electrode of the MFCs had a carbon veil sheet (10 g.*m* ^−^ ^2^; Technical Fibre Products Ltd, Cumbria, UK) with a stainless-steel wire that was operating as the current collector. The 150 cm^2^ carbon veil was loaded (7.3 ± 0.4 mg*_AC/PTFE_*.cm^−2^) with an AC/PTFE (activated carbon (AC); polytetrafluoroethylene (PTFE)) mixture (80 wt% AC; 20 wt% PTFE) [24] and folded down to a geometric surface area of 6.25 cm^2^. The anodic chamber had a total volume of 10 mL. As previously described [25], the cathodes consisted of the same AC/PTFE mixture pressed on a stainless-steel 316 mesh acting both as a support and current collector. The AC/PTFE loading on the cathode was of 186 ± 7 mg*_AC/PTFE_*.cm^−2^.

All MFCs were identical in geometry and structural material except for the ceramic membrane.

### 2.3 Microbial fuel cell operation

The experiment analysed the performance of the MFCs depending on both the iron oxide content and the microstructural porosity features. The MFCs were inoculated with 50% partially hydrolysed urine and 50% in volume of effluent from running MFCs. After leaving the MFC in open circuit condition for 12 h, they were connected to an external device that was maintaining constant the operating voltage at 380 mV [26]. The output by the device was monitored by an Agilent Data Acquisition System (Agilent LXI 34972A; Farnell, UK) logging data every 2 min. The acquired voltage is an indirect measurement of the current and the following formula was used to calculate current:

(1) $$I=\frac{\left(V_{m}-1.20\right)}{19.8}$$

Where *I* is the current in Amperes (A) and *V_m_* is the voltage measured in Volts (V) by the acquisition system (i.e. Agilent 34972A). The value 1.2 is the offset voltage applied to the circuitry, which corresponds to the measured voltage when nothing is connected to the circuit, whereas 19.8 is the conversion factor of the microchip (in mV/mA) that is used to convert the current reading into a voltage. The power *P* in Watts (W) produced by each unit was calculated using the formula, *P = I x V*, where *V* is the constant voltage (380 mV) in Volts (V) and *I* is the calculated current using Eq. (1).

After inoculation, the MFCs were fuelled with partially hydrolysed urine that was collected daily from a tank pooling together the urine donated by anonymous healthy individuals (pH of 8.8–9.2, solution conductivity of 27–30 mS.cm^−1^). The fuel was pumped (peristaltic pump, Watson & Marlow LTD, UK) at a flow rate of 3.5 ml.*h* ^−^ ^1^, resulting in a hydraulic retention time of 2.86 h for each MFC. After inoculation, the 9 different conditions were tested simultaneously for roughly 800 h (including the maturing stage), then the ceramic membranes were swapped for new membranes having the same characteristics (swapping “like for like”) until completing the triplicate tests. Overall, all conditions were tested three times (the first one for 800 h and second and third for 400 h).

### 2.4 SEM-EDX membrane characterisation

Small specimens of ceramic membrane samples were mounted by embedding them within low viscosity epoxy resin (EpoFix, Struers, Denmark) under vacuum (CitoVac chamber, Struers, Denmark) to fill in the inner porosity voids and to preserve microstructures of porous membranes. Cured mounts were ground to expose transversal cross-sections of membranes and were subsequentially polished ending with 3 mm diamond paste.

The obtained membrane material samples were analysed with scanning electron microscopy (SEM) and energy dispersive X-ray (EDX) spectroscopy (JEOL 6010PLUS/LA) with an acceleration voltage of 20 kV in secondary (SE) and backscattered electron (BSE) modes.

### 2.5 Porosity and pore size distribution analysis

The microstructural features of the ceramic membranes were characterised in terms of total effective porosity (hereafter referred to as “porosity”) and pore size distribution by using mercury intrusion porosimetry (MIP), which is a widely used technique for analysing both parameters in a broad range of materials [27]. To this end, a Poremaster-60 GT (Quantachrome Instrument, United Kingdom) was used, which was equipped with dual high-pressure transducers to enhance accuracy across the entire analytical range. The device also includes two built-in automated low-pressure ports for filling of penetrometers and intrusion/extrusion measurements from vacuum to 60.000 psi. Before performing the measurements, the samples were dried at 60 °C overnight and then, placed under vacuum. The range of pressure for the intrusion measurements was 6.5 – 408 167.6 KPa, while the extrusion measurements were performed between 405 003.5 - 138.4 KPa.

### 2.6 Electrochemical characterisation of microbial fuel cells

Before running the electrochemical characterisation experiments, the MFCs were disconnected from the constant load device and maintained under open-circuit voltage (OCV) condition for 1 h. The polarisation experiments were performed running linear sweep voltammetry (LSV) in a two-electrode configuration (potentiostat biologic SP-50) with the reference electrode channel short-circuited with the counter electrode channel, the cathode acting as the working electrode and the anode serving as the counter electrode. The scan rate was 0.25 mV.*s* ^−^ ^1^ and ranged from OCV to 25 mV.

### 2.7 Statistical analysis

The significance of the variables (sintering temperature and iron content) on the power performance of MFCs was statistically analysed by using the analysis in variance model (ANOVA). Then, Tukey's HSD post hoc analysis was used to find means that are significantly different from each other. The statistical analysis was developed by using Spyder 3.3.6, a development environment for scientific programming in Python (Python 3.7).

## 3 Results and discusion

### 3.1 Membrane characterisation

**Fig. 2:**
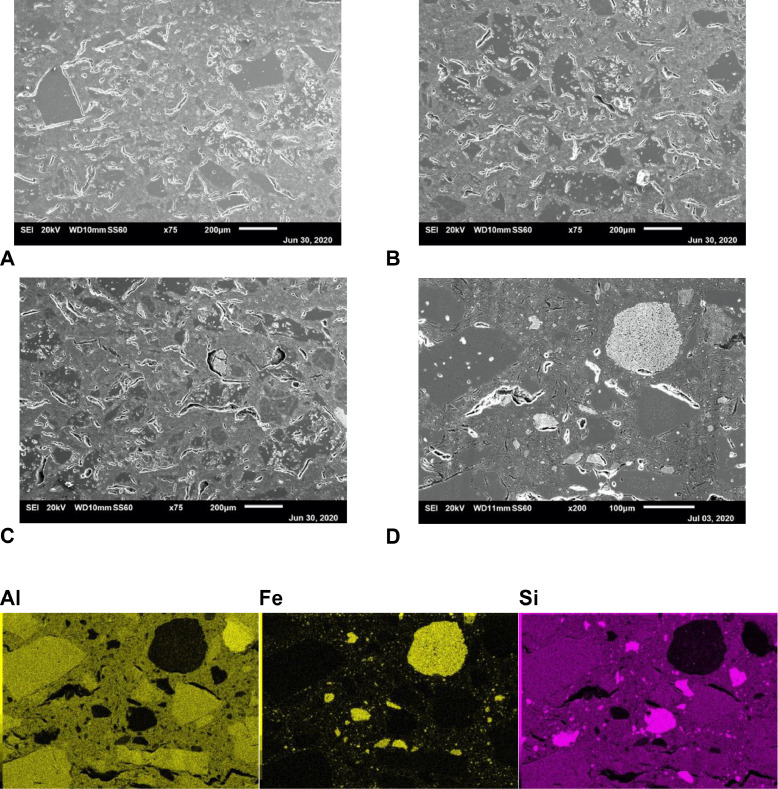
SEM micrographs corresponding to polished cross-sections of ceramic membranes: A) F1-T1, B) F3-T1 and C) F6-T1. Higher magnification SEM micrograph of F6-T1 membrane (D) with corresponding EDX mapping of main chemical elements.

Fig. 2 shows polished cross-sections of ceramic membranes with varying iron oxide content sintered at 1100 °C. Low magnification micrographs show microstructures with large grain distribution for all three compositions (Fig. 2A, B and C), a typical feature for refractory clay materials. Relatively low sintering temperature used in this case resulted in highly interconnected porosity without the need to deploy sacrificial pore-forming agents. Low densification effect is highlighted by sharp edges of some grains and poor intergranular fusion. Higher magnification micrograph corresponding to F6-T1 ceramic membrane cross-section is shown in Fig. 2D with corresponding EDX elemental mappings below. As can be seen , the sharp edge grains (corresponding to the filler function of refractory ceramic material) are mainly composed of aluminium oxide, whereas silica oxide performs the main matrix binding function. The iron oxide phase is evenly distributed within the main matrix binding phase with the sporadic presence of large porous agglomerates (reaching up to 100 μm diameter range). The same compositional features were observed by EDX for F1 and F3 compositions employed in the study (not presented here). SEM microstructural observation and EDX compositional mapping confirmed the absence of detrimental effects within the refractory pastes studied, such as the formation of secondary phases or phase evaporation.

**Fig. 3:**
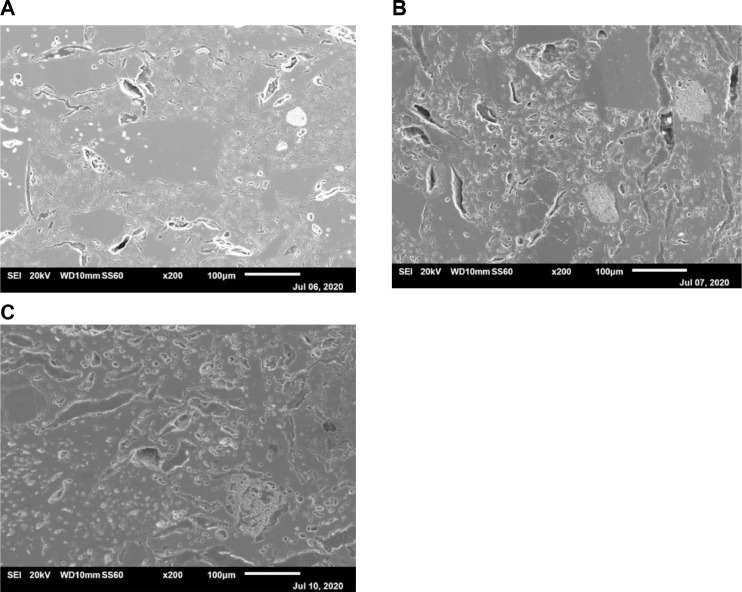
SEM micrographs corresponding to polished cross-sections of F3 composition ceramic membranes: A) F3-T1, B) F3-T2 and C) F3-T3.

Fig. 3 shows polished cross-sections of ceramic membranes with fixed iron oxide content (F3 composition) sintered at temperature range 1100–1300 °C. A strong effect of sintering temperature on microstructural features can be observed, where the aluminium oxide phase merging with the main matrix phase with increasing temperature is reached. As a sequence, pore microstructure is shifted from mainly open pore network (Fig. 3A) to finer entrapped pore network. Nevertheless, the large elongated pores formed due to the large particle size of starting pastes is preserved to some extent even at the highest sintering temperature (Fig. 3C). It is interesting to note that fine porosity features observed in iron oxide phase agglomerates were also preserved .

**Fig. 4:**
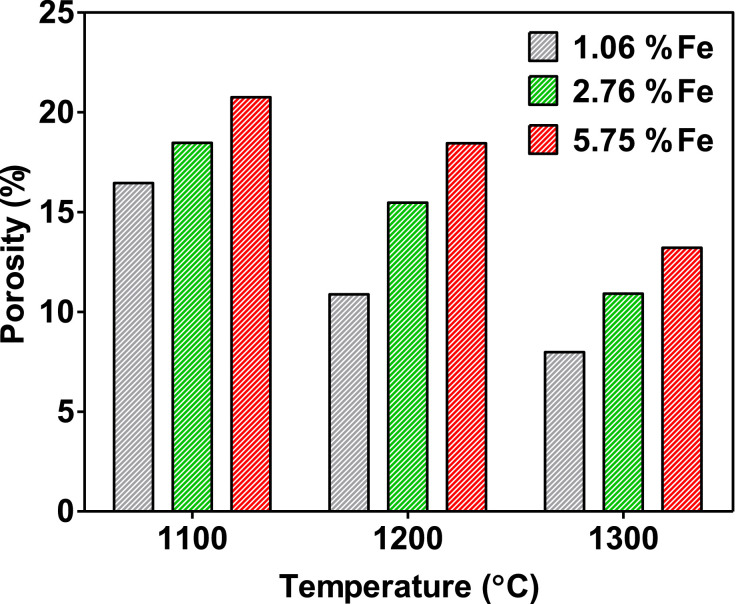
Porosity of the ceramic membranes elaborated from clays with varying iron oxide content.

Fig. 4 summarises the data obtained by MIP corresponding to the porosity of the different ceramic membranes elaborated. As can be seen, sintering temperature and compositional variation or raw ceramic clays have a significant effect on the porosity features of the membranes produced. The porosity of the ceramic membranes was increasing with the increasing amount of the iron oxide phase for all compositions at a given sintering temperature. As expected, there is a clear trend for porosity reduction with an increase in sintering temperature for all-ceramic clay compositions. This is in good agreement with previous work performed in similar MFC set-ups [28]. The maximum porosity value measured was 20.75% for membranes with the highest amount of iron oxide phase and sintered at the lowest temperature. By contrast, the minimum value of porosity was exhibited for ceramic membranes containing the lowest amount of iron oxide phase and sintered at the highest temperature (7.98%).

**Fig. 5:**
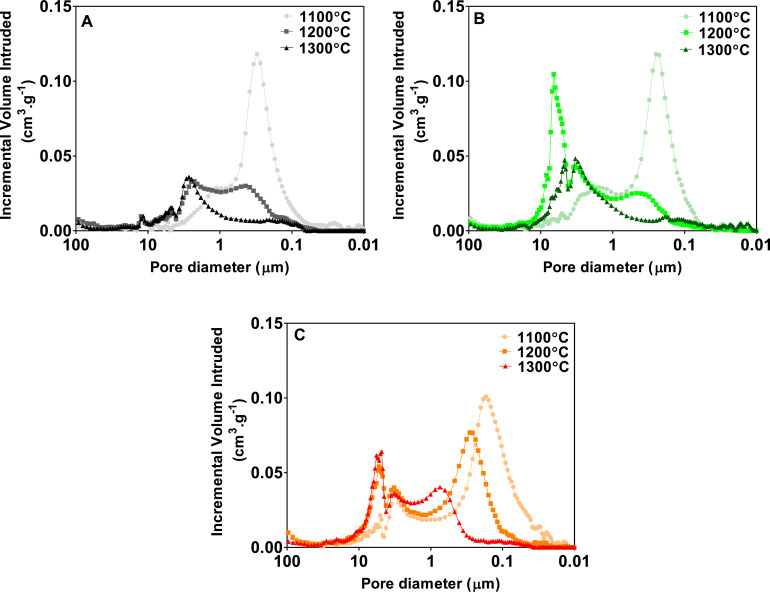
Pore size distribution of the different ceramic membranes studied; A) 1.06%, B) 2.76% and C) μ5.75% of iron oxide content.

Fig. 5 depicts the pore size distribution data from the MIP analysis of the membranes elaborated. The data show that regardless of the amount of iron oxide, all membranes elaborated show a bimodal pore size distribution and that the pore size increases with increasing sintering temperature [28]. That is, for all compositions, the surface area of the right peak (belonging to smaller pore size) decreases as the sintering temperature increases, whereas the area of the left peak (belonging to larger pore size) increases. The effect of gradual coarsening with increasing temperature is very clear for 5.75 vol.% iron oxide composition (Fig. 5C) where two main peaks of the bimodal pore size distribution are gradually shifted to the left side of the plot. For instance, 70.45% of the pores in the membranes containing 5.75vol. % of iron oxide and sintered at 1100 °C showed a size between 1.08 and 0.0373 µm, whereas 19.81% of the pores have a size ranging between 15.91 and 1.08 µm. However, membranes with the same amount of iron oxide but sintered at 1200 °C showed 51.78% of the pores between 1.068 and 0.0961 µm, while the 35% of the size of the pores ranged between 16.07 and 1.068 µm. Finally, when these membranes were sintered at 1300 °C, 27.47% of the pores were between 1.063 and 0.2575 µm, whereas 59.08% of the pores have a size between 16.02 and 1.063 µm. Similar behaviour was observed in the pore size distribution of membranes containing 1.06 and 2.76% of iron oxide.

For a specific temperature, the compositions of clays with increasing iron oxide content resulted in microstructures with decreasing pore size distribution. The observed enlargement of the pore size with increasing sintering temperature is due to the microstructural coarsening (coalescence of smaller microstructural features such as grains and pores) resulting in coarser and overall denser material. The absence of liquid phase sintering process for a given temperature range (1100–1300 °C) indicates that ceramic clays employed in this study had negligible amounts of carbonates [29,^30^,^31^,32].

Rago et al. (2018) [33] reported that pore size between 0.1 and 0.01 μm in terracotta membranes hinder the development of a biofilm layer onto the air-cathode surface, which limits the current production. By contrast, the presence of the terracotta membranes in MFCs barely affects the electroactive anodic communities compared to the membrane-less reactors. In the present work, the pore size of most of the samples is above 0.1 μm which might promote the development of a biocathode, improving the abiotic catalytic activity for oxygen reduction and therefore, the power output.

**Fig. 6:**
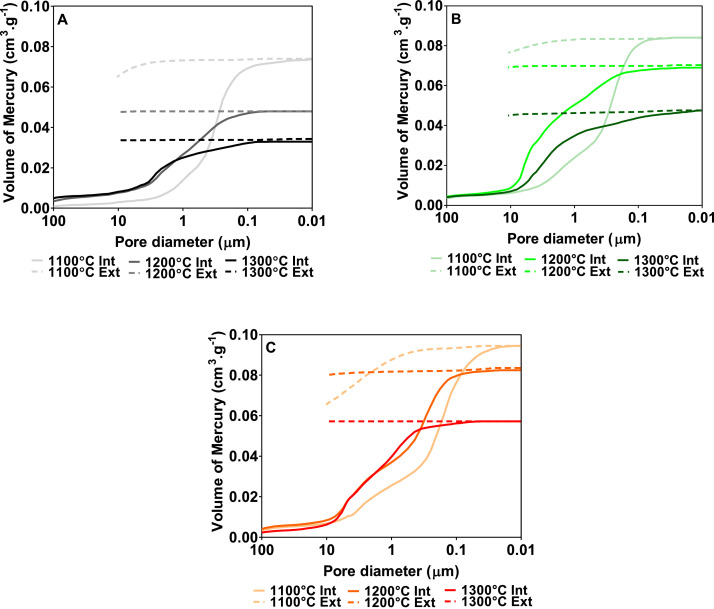
Volume of mercury intruded (solid line) and extruded (dashed line) in the porous surface of the different iron composite ceramic membranes tested: A) 1.06%, B) 2.76% and C) 5.75% of iron oxide content.

Fig. 6 shows the cumulative volume of the mercury intruded and extruded in the porous surface of the elaborated ceramic membranes with varying iron oxide content. As can be seen, regardless of the iron oxide content, the volume of mercury intruded decreases as the sintering temperature increases, being higher for ceramic membranes elaborated at 1100 °C and lower for ceramic membranes made at 1300 °C. Regarding the effect of the varying initial clay composition, it was observed that the volume of mercury intrusion increases for compositions with an increasing amount of iron oxide phase. These results are in line with the porosity results previously discussed, the ceramic membranes with a higher percentage of porosity being those in which the volume of mercury intrusion is higher. On the other hand, it can also be observed that the extrusion curve does not follow the intrusion curve and therefore, the intrusion/extrusion cycle is not closed when the process goes back to the initial pressure. The difference between the volumes of mercury intruded and extruded means that part of the mercury is trapped within the pores of the ceramic microstructure when the process finishes. This difference is caused by the presence of not only an intricate pore network but also pore bottleneck effects [31,^34^,35]. It is worth highlighting that the extrusion curve for membranes sintered at 1200 and 1300 °C is almost flat which confirms that most of the mercury intruded kept inside of the porous structure of the membrane when the process analysis finished . Only membranes elaborated at 1100 °C show slightly higher volume of mercury extruded, recording a maximum for membranes containing 5.75vol. % of iron oxide (see Fig. 5C).

### 3.2 Microbial fuel cell performance

**Fig. 7:**
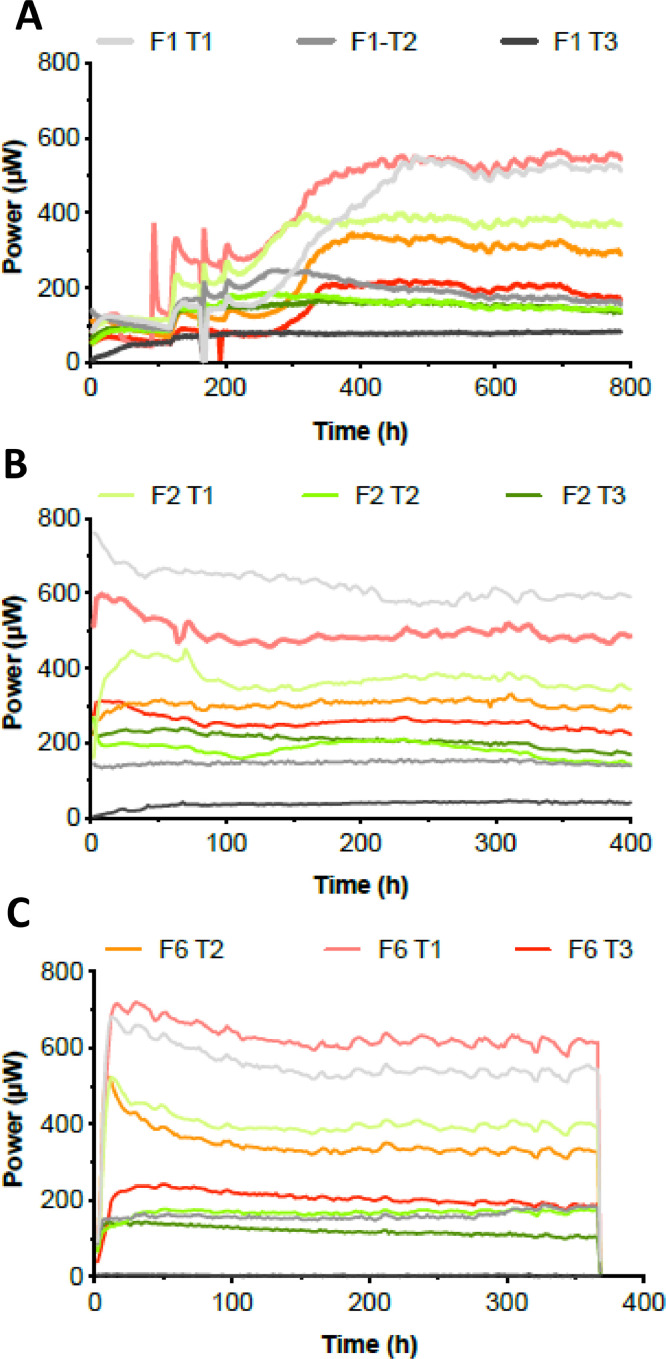
Power output by the MFCs working with the different membranes. All different conditions were tested simultaneously whilst each triplicate of the tested conditions was run sequentially and are plotted separately (A, B and C plots).

Before characterising the electrochemical performance, the stability of the electrical output was investigated for a duration of 400 h for each of the tested conditions and each of the triplicates. As explained in the Material & Methods section, each of the triplicates was tested sequentially by only replacing the membrane of the working MFC every 400 h. As shown in Fig. 7A, the inoculation phase was of roughly 400 h prior to the MFCs displayed stable power outputs. With the exception the F1-T1 condition (450 h), the results show that the MFCs with iron-rich membranes (5.75vol. %) were the last ones to reach steady states (roughly 350 h; Fig. 7A). The results also indicate that all tested conditions had similar power output levels between triplicates, prior to the main tests (Fig. 7A, B, C), thus demonstrating that the observed variations were indeed due to the membrane functionalisation. The main observation is that all the MFCs equipped with membranes sintered at 1100 °C displayed higher power output levels, independently from the triplicate (Fig. 7A, B, C) or the iron content (Fig. 7). Moreover, once a steady state was reached, the iron-rich membranes (5.75vol. %) were displaying the highest power outputs, with respect to the sintering temperature. Interestingly, the MFC mounted with the membranes having the lowest iron content and sintered at the lowest temperature (i.e. F1-T1) and the MFC equipped with the membranes having the highest iron content and sintered at the lowest temperature (i.e. F6-T1) displayed a somewhat similar electrical output that was always the highest compared to the other conditions. However, the differences in power output between the iron-rich and iron-poor membrane increased with increasing sintering temperatures. Although all current output levels decreased with increased sintering temperature, the more iron the membrane contained the least they were affected by the temperature increase. This is particularly true for the membrane containing 1.06% iron and sintered at 1200 °C (F1-T2) that showed for each triplicate an average 71±5% power output decrease compared to the F1-T1 condition. Conversely, the F6-T2 condition displayed an average 42±4% power output decrease compared to the F6-T1 condition. These results illustrate the “buffering capacity” in performance losses, toward increased sintering temperature, offered by iron-rich membranes. This result has to be placed in the light of where these ceramic MFCs would be deployed, in decentralised areas of countries with a developing economy where ceramics are kilned using wood fire with less temperature-control. In such a context, the addition of iron in the ceramic composition would ensure better material stability of the membrane to the temperature variations found in this fire-driven kiln.

**Fig. 8:**
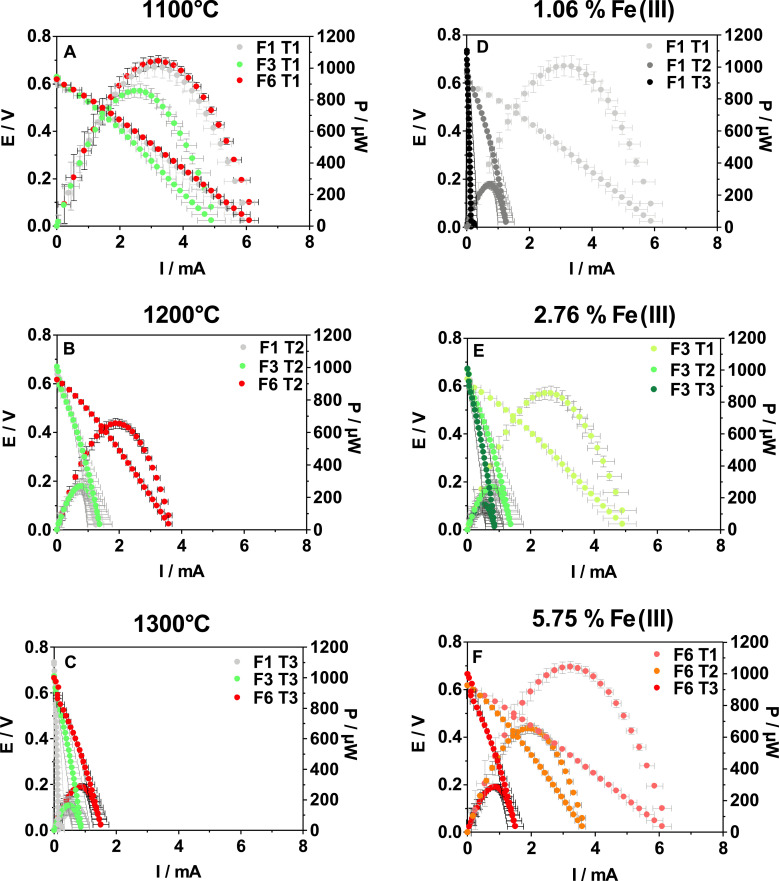
Polarisation and power curves of the MFCs working with the different membranes elaborated.

Fig. 8 displays the average electrochemical performance of all cells when they reached the steady-state. According to the long-term current output by the systems previously discussed, the sintering temperature negatively affects the power performance of the MFCs so the maximum power output decreases as the sintering temperature increases. However, it was also observed that the presence of iron reduces the effect of the temperature on the power output by the MFCs. For instance, the reduction in power observed when the membranes containing 1.06% of iron were sintered at 1200 °C and 1300 °C instead of 1100 °C was 73.7% and 96.9%, respectively. However, when the amount of iron was increased up to 2.76%, the reduction in power for those temperatures was 68.6% and 80.9%, respectively. Finally, when the presence of iron was increased up to 5.75%, the reduction in power observed for the same temperatures was 37.3% and 72.6%, respectively. These results are in accordance with those displayed by the temporal runs (Fig. 7). According to these results, the MFCs reached the highest values of power output when they worked with membranes containing the highest amount of iron, regardless of the sintering temperature. Thus, the maximum power output by MFCs was 1045.3, 655.8 and 286.6 µW when they worked with membranes sintered at 1100, 1200 and 1300 °C containing 5.75vol. % of iron, respectively. These results might be related to the increase in the ion exchange capacity and permselectivity of the membranes due to the presence of Fe_2_O_3_ in their internal structure. In addition, a reduction in electrical resistance of membranes containing Fe_2_O_3_ has also been reported [36], which enhances the MFC power output. Namdari et al. [36] recently reported that the amount of sulphated Fe_2_O_3_ particles in the membrane matrix enhances its ion exchange capacity whilst the dispersion of the particles remains uniform. For high amounts of Fe_2_O_3_ particles, it is crucial to keep the particles uniformly dispersed to avoid forming aggregates, which reduce the ion exchange capacity, the transport rate and the permselectivity of the membrane. In the present work, Fe_2_O_3_ particles are uniformly integrated into the structure of the membranes instead of dispersed onto the surface which avoids this problem. These findings are in line with those reported in the present work. The use of Fe_2_O_3_ to modify both polymer and ceramic membranes results in an easy way to improve the membrane properties in terms of not only ion transfer but also catalytic activity. When Fe_2_O_3_ nanoparticles are used to modify ceramic materials, the number of nanopores in the structure increases, which promotes their adsorption and catalytic properties to degrade pollutants under specific conditions [37,38]. These modifications of the membrane structure result in the development of selectively functionalised structures for specific purposes, which improved efficiency.

Taking into account the different sintering temperatures studied, the power performance of the MFCs improved when the membranes contained the highest amount of iron, for each temperature investigated. Thus, the addition of 5.75% of iron instead of 1.06% for membranes sintered at 1100 °C allowed MFCs to enhance the power output up to 3.7%. However, at 1200 °C the improvement of the power output reached 147.5% when comparing the 1.06% and the 5.75% conditions (from 265.0 µW to 655.8 µW, respectively). However, for the same increase in the amount of iron, the percentage of power output improvement was 147.5%, when the membranes were processed at 1200 °C (from 265.0 up to 655.8 µW). Finally, when the iron concentration increased from 1.06 to 2.76%, for membranes sintered at 1300 °C, the percentage of power output improvement observed was 430.5% (30.9 and 164.1 µW, respectively) whereas this value increased almost two times when the amount of iron increased from 2.76 up to 5.75% (maximum power output of 286.6 µW). As previously discussed, the presence of the higher amount of iron in the structure of the membranes, for the different sintering temperatures investigated, increases the porosity of the membranes as opposed to the membranes with a lower amount of iron and elaborated at the same temperature. This increase in porosity might benefit the ion exchange through the membrane and therefore the power performance of the MFCs. The use of high sintering temperatures reduces the porosity of the membranes which increases their bulk resistance and therefore, reduces the power performance of the MFCs [39,28]. The results obtained in this work show that the presence of iron in the internal structure of the membrane helps to mitigate the effect of the temperature on the membrane porosity and therefore, on the power output by the system.

Cheraghipoor et al. 2019 [40] also reported that membranes made of leached Kalporgan's soil (LKS) with 4.52% of Fe_2_O_3_ allowed them to improve the power performance of their cylindrical double-chamber MFCs up to 42 times compared with the system working with membranes based on commercial ceramic powder containing 2.92% of iron, which is in line with the results obtained in the present work.

You et al. 2019 [41] analysed the effect of different type of ceramic materials on the power performance of urine-fed MFCs. Amongst the different materialsreported, the MFCs working with Red ceramic as a membrane outperformed those working with White ceramic (670.5 and 570.5 µW, respectively). Both ceramic materials were elaborated at high temperature, ranged between 1070 and 1240 °C, but the iron content was different, 10.3% in the case of the Red ceramic and 2.7% for the White material. These results are in good agreement with those reported in the current work, since the power output by the MFCs improved as the iron content in the ceramic membranes increases, for high firing temperatures.

**Fig. 9:**
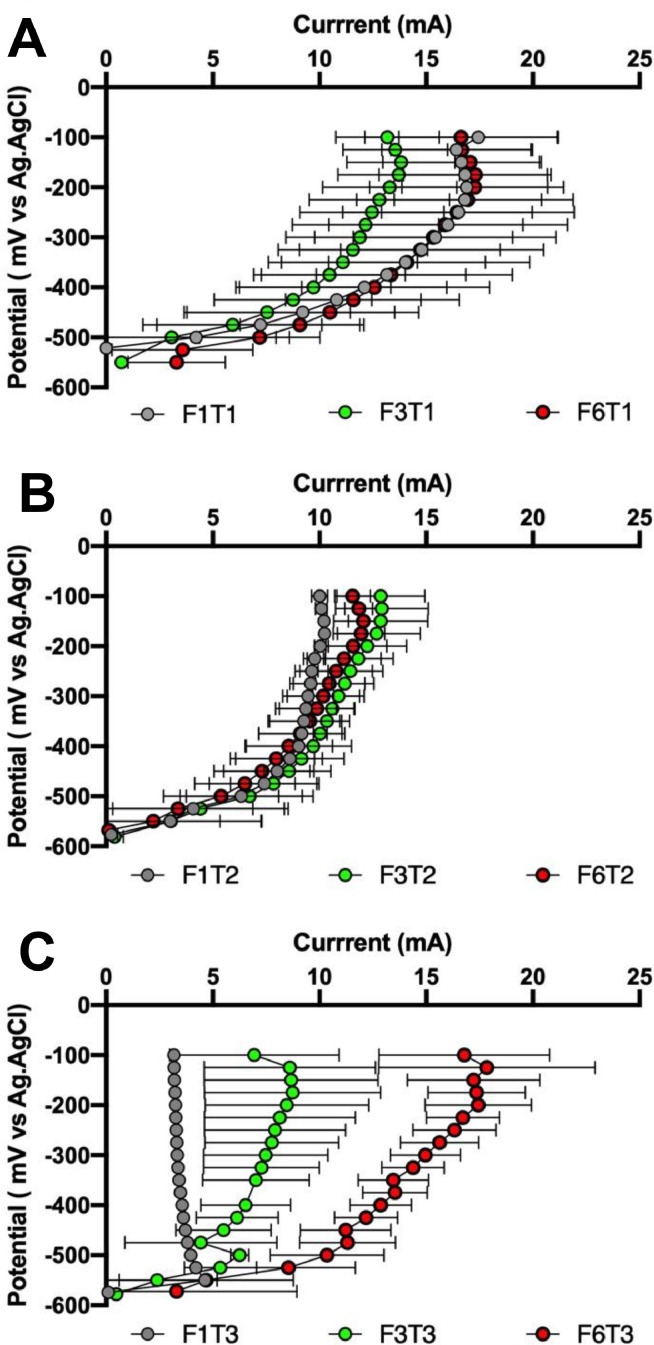
Anode polarisation of the MFCs working with the different membranes elaborated. A) *T* = 1100 °C, B) *T* = 1200 °C and C) *T* = 1300 °C.

**Fig. 10:**
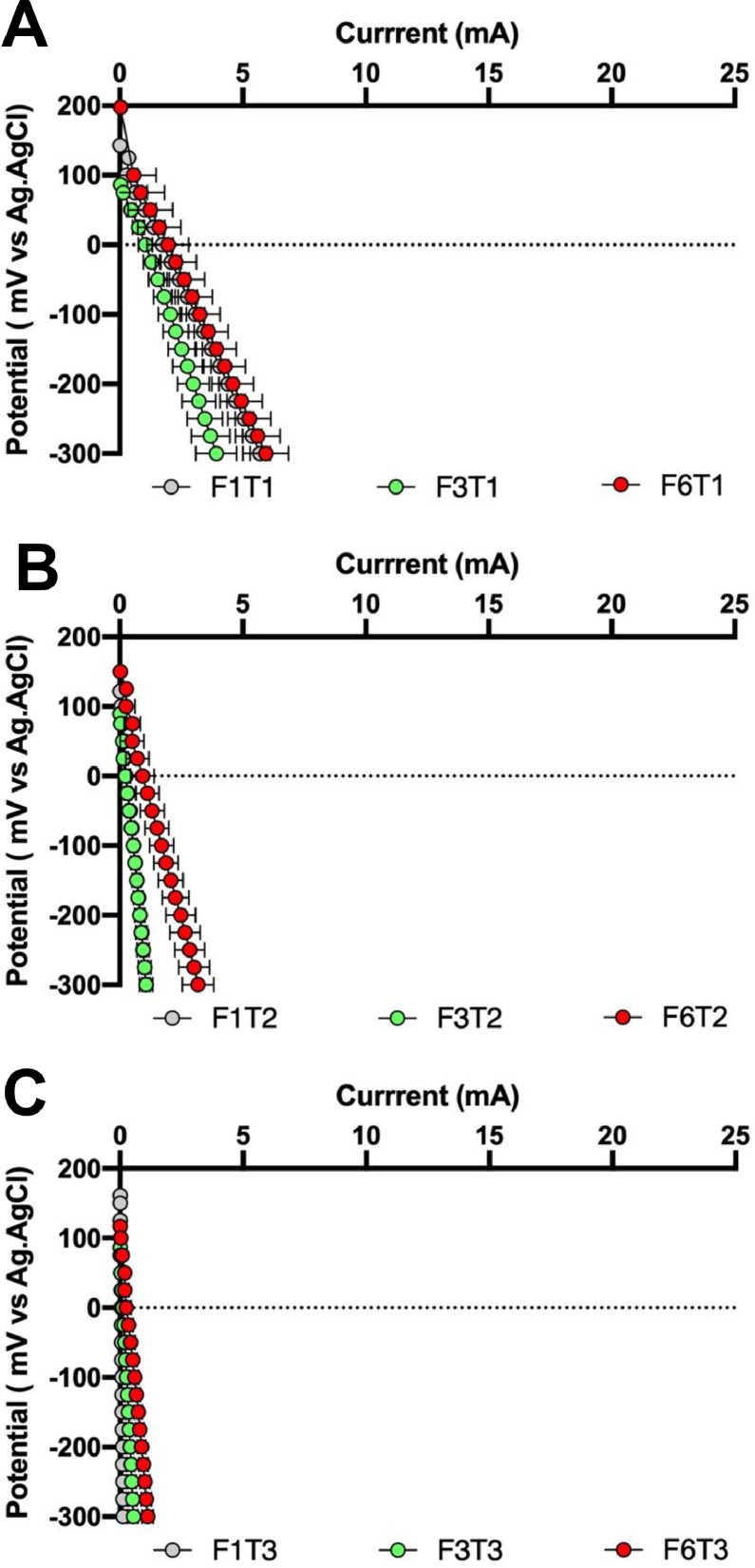
Cathode polarisation of the MFCs working with the different membranes elaborated. A) *T* = 1100 °C, B) *T* = 1200 °C and C) *T* = 1300 °C.

As can be seen in Fig. 9, Fig. 10, the results of the individual electrode polarisation showed a similar trend. An increase in the sintering temperature of the membranes results in a reduction of the cathode efficiency. These results may be related to the reduction in porosity observed as the temperature increases, which hinders the formation of a cathodic biofilm. This reduction in performance observed in the cathode, when the sintering temperature increases, seems to be mitigated by the presence of Fe_2_O_3_. Regarding the anode, the development of an electroactive biofilm seems to be promoted by the presence of Fe_2_O_3_ in the membrane, since the anodes perform better as the percentage of Fe_2_O_3_ increases, regardless of the sintering temperature. The enhancement of the permselectivity and ion transport of the membranes due to the presence of Fe_2_O_3_ might help to develop an efficient anodic biofilm, which directly results in higher power performance of the MFCs.

### 3.3 Statistical analysis

A linear regression model was fitted to the data (Table S1 in Supplementary Material). The Jarque-Bera and Omnibus test results allowed us to verify the normality and homogeneity of the variance, respectively, whereas the results of the Durban-Watson test and the Condition number dismissed the presence of autocorrelation and multicollinearity, respectively. Therefore, the model passed all the assumption checks for the ANOVA model.

Table 3 provides the ANOVA model for the effect of the input variables, sintering temperature and iron content, on the MFCs power performance. The results show that both parameters, as well as the interaction between them, have a significant effect with 95% confidence (*p*<0.05) on the power output.

**Table 3:** Analysis of variance (ANOVA) for the effect of the sintering temperature and iron content on the MFCs power performance.

ANOVA ANALYSIS					
Index	**Df**	**Sum_sq**	**Mean_sq**	**F**	**PR(>*F*)**
C (Fe)	2	317,236	158,618	90.2611	4.14164 × 10^−10^
C (Tª)	2	3.12142 × 10^6^	1.56071 × 10^6^	888.116	1.0293 × 10^−18^
C (Fe): C (Tª)	4	141,336	35,334.1	20.1068	1.91128 × 10^−6^
Residual	18	31,631.9	1757.33	–	–

Table S2 and S3 (see supplementary material) show the Tukey's HSD test for the iron content and temperature, respectively. As previously commented, the post hoc test is used to find means that are significantly different from each other. The results show that despite the iron content has a significant effect on power performance, there are no significant differences amongst the different amounts of iron studied. It means that the presence of iron in the membrane structure affects the power performance but any of the amounts studied prevails over others (see Table 3). However, in the case of the temperature, it was observed that the different values of temperature studied has a statistically significant effect on the power output from the MFCs according to the following trend: T1>T2>T3. These results support that the highest the sintering temperature, the lowest power output.

## Conclusions

The aim of this work is to evaluate the effect of the iron content and the sintering temperature of ceramic membranes on the power performance of urine-fed MFCs. To this end, three different amounts of iron (1.06, 2.76 and 5.75vol. %) and three different sintering temperatures (1100, 1200 and 1300 °C) were combined to fabricate 9 different types of membranes, which were tested in triplicate. The results show that the presence of iron not only increases the porosity of the membranes but also reduce the pore size. It is worth highlighting that the presence of iron in the membranes mitigates the negative effect of high sintering temperatures on the power output by the MFCs. Thus, the increase of iron content from 1.06 up to 5.75vol. % allowed MFCs to reach a maximum power output that was ca. 10-fold higher (30.9 and 286.6 µW, respectively). Amongst the different combinations of iron content and sintering temperatures, the maximum power output was obtained by MFCs working with membranes containing 5.75vol. % of iron and kilned 1100 °C (1.045 mW). The functionality of the different membranes tested was evaluated for 65 days, which confirms the suitability of the different materials analysed for long periods of work, very common in practical applications.

## Credit authorship contribution statement

**MJ. Salar-García, X. A. Walter and J. Gurauskis:** Conceptualisation, Methodology, Data curation, Formal analysis, Investigation, Methodology, Visualisation, Writing - original draft, Writing - review & editing. **A. de Ramón Fernández:** Formal analysis, Writing - original draft, Writing - review & editing. **I. Ieropoulos:** Conceptualisation, Funding acquisition, Project administration, Resources, Supervision, Writing - original draft, Writing - review & editing.\

## Declaration of Competing Interest

The authors declare that they have no known competing financial interests or personal relationships that could have appeared to influence the work reported in this paper.

## Appendix Supplementary materials

Image, application 1
