# Supplementary material for: Effect of iron oxide content and microstructural porosity on the performance of ceramic membranes as microbial fuel cell separators
Source: Electrochim Acta. 2021 Jan 20;367:137385. doi: 10.1016/j.electacta.2020.137385 (PMC7829595; doi:10.1016/j.electacta.2020.137385)
Supplement: Supplementary file 1 [file mmc1.docx]

**SUPPLEMENTARY MATERIAL**

Table S1. Ordinary least squares regression model.


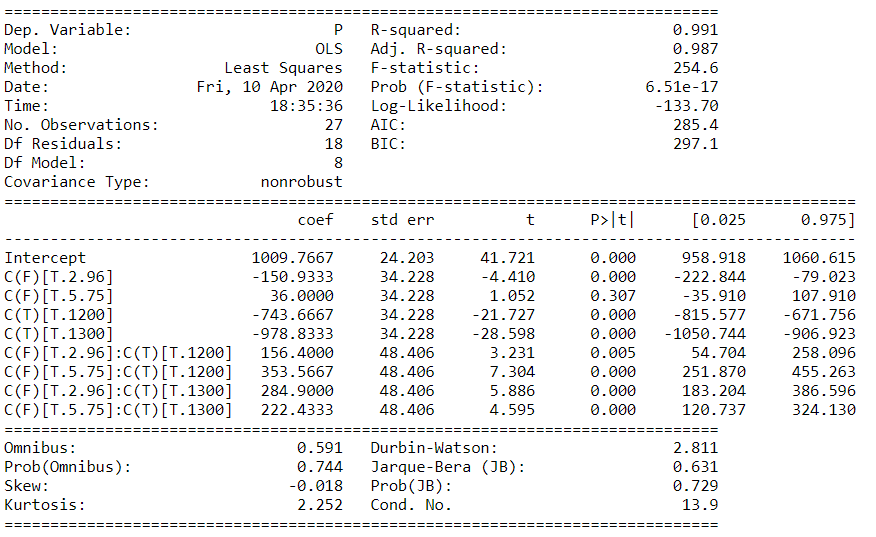


Table S2. Post Hoc analysis for the iron content.


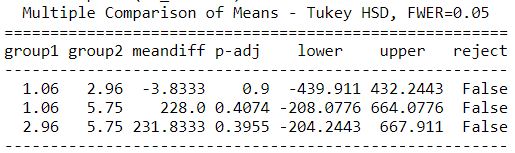


Table S3. Post Hoc analysis for the temperature.**
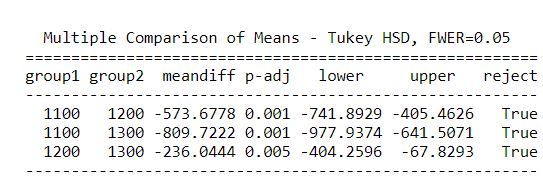
**
